# Phytochemical Analysis and Bioactivities of *Dombeya rotundifolia* and *Lippia javanica*

**DOI:** 10.3390/plants15142233

**Published:** 2026-07-22

**Authors:** Matsilane L. Mashilo, Mashilo M. Matotoka, Ofentse Mazimba, Peter Masoko

**Affiliations:** 1 Faculty of Science and Agriculture, Department of Biochemistry, Microbiology and Biotechnology, University of Limpopo, Private Bag X1106, Sovenga 0727, South Africa; lee.mashilo79@gmail.com (M.L.M.); mashilo.matotoka@ul.ac.za (M.M.M.); 2 Chemical and Forensic Sciences, Botswana International University of Science and Technology, Private Bag 16, Palapye 10017, Botswana; mazimbao@biust.ac.bw

**Keywords:** antimycobacterial, antioxidant, anti-inflammatory, *Dombeya rotundifolia*, *Lippia javanica*

## Abstract

Tuberculosis (TB) remains a major global health challenge, compounded by rising drug resistance. Traditional medicinal plants used for TB-related symptoms represent a valuable yet underexplored source of potential antimycobacterial agents. In this study, the phytochemical content and biological activities of *Dombeya rotundifolia* and *Lippia javanica* were evaluated, and antimycobacterial compounds were isolated through bioassay-guided fractionation. Plant leaves were collected, extracted with solvents of varying polarity, and screened for phenolics and flavonoids. Antioxidant activity was assessed using DPPH and ferric reducing power assays, antimycobacterial activity was tested against *Mycobacterium smegmatis*, anti-inflammatory activity was determined by the egg albumin denaturation method, and cytotoxicity was evaluated in THP-1 cells using the MTT assay. Extraction yields varied, with water extracts of *L. javanica* showing the highest yield and hexane extracts of *D. rotundifolia* the lowest. Both plants contained bioactive phytochemicals with measurable antioxidant activity, while acetone and dichloromethane extracts of *D. rotundifolia* displayed the strongest antimycobacterial activity with MIC values of 0.16 mg/mL. Cytotoxicity assays indicated moderate toxicity at higher extract concentrations. Bioassay-guided isolation led to the identification of two fatty acid fractions with tentative characterization, which showed notable antimycobacterial activity (MIC 0.25 mg/mL). These findings provide preliminary support for the ethnomedicinal use of *D. rotundifolia* in respiratory conditions traditionally associated with tuberculosis and suggest a potential contribution of its fatty acids to its observed antimycobacterial activity.

## 1. Introduction

Tuberculosis (TB), caused by members of the *Mycobacterium tuberculosis* complex, remains a leading global health challenge, with an estimated 10.6 million new cases and 1.6 million deaths reported in 2021 [1]. Rising incidence and the emergence of multidrug-resistant (MDR) and extensively drug-resistant (XDR) strains have intensified the search for novel therapeutic agents [2]. Current first-line drugs such as isoniazid, rifampicin, pyrazinamide, and ethambutol are increasingly compromised by resistance and are often associated with severe side effects and interactions with antiretroviral therapies [3,4].

*Mycobacterium smegmatis* has become an important surrogate model for TB research owing to its rapid growth, genetic tractability, and physiological similarities to pathogenic mycobacteria. It has been instrumental in drug discovery, including the development of bedaquiline, and continues to provide valuable insights into resistance mechanisms and drug targets [5,6,7].

Plants have long served as sources of bioactive compounds with antimicrobial properties. Phytoconstituents such as alkaloids, flavonoids, tannins, phenolics, sterols, and terpenoids exhibit antimycobacterial activity and may offer safer, complementary approaches to TB management [8,9]. *Dombeya rotundifolia* (Hochst.) Planch, known as wild pear (Malvaceae family), is traditionally used for treating stomach disorders, fever, chest complaints, and pneumonia. Different parts of the plant, including the leaves, bark, shoot, flowers, woods and stems, are commonly used in traditional medicine, and their extracts have demonstrated antioxidant, antimicrobial, and anti-inflammatory effects [10,11]. *Lippia javanica* (Burm.f.) Spreng (Verbenaceae family), commonly used as herbal tea, while multiple parts of the plant, such as the leaves, roots, twigs, and stems are employed in the treatment of coughs, colds, TB, and related respiratory ailments, with reported antioxidant, antibacterial, and antiviral properties [12,13]. The leaf was reportedly used in the treatment of TB [14]. The following authors reported antimycobacterial activity of leaf acetone extract against *M. smegmatis* [15], and a compound isolated from ethanol extract of *L. javanica* was found to be effective against *M. tuberculosis* [16].

Among the diverse phytochemicals found in these plants, recent studies have shown that specific fatty acids possess significant antimycobacterial activity. Myristic acid was the most active, with an MIC of ~10 µg/mL against *Mycobacterium bovis* (Ravenel) and *M. tuberculosis* H37Ra [17]. Lauric acid (C12:0) exhibited MICs of 6.25–25 µg/mL, while capric acid (C10:0) showed 50–100 µg/mL against *M. tuberculosis* [18]. In *Citrus aurantiifolia* fruit peel extracts, palmitic (25–50 µg/mL), linoleic (50–100 µg/mL), and oleic acids (100 µg/mL) demonstrated activity against *M. tuberculosis* H37Rv and multidrug-resistant strains [19].

In addition to fatty acids, phenolic compounds have been widely recognized for their antimycobacterial potential. These compounds may directly inhibit bacterial cell division, while flavonoids, a major subclass of polyphenols, interfere with essential enzymatic pathways and disrupt mycobacterial cell wall assembly [20]. Several polyphenols, including epicatechin, kaempferol, quercetin, luteolin, rutin, and myricetin, have demonstrated activity against non-tuberculous mycobacterial species such as *M. smegmatis*, *M. fortuitum*, and *M. phlei*. Additionally, compounds such as epigallocatechin gallate, curcumin, resveratrol, and quercetin have shown inhibitory activity against *M. tuberculosis* [21]. The investigation of these phytochemicals is important in tuberculosis research because they represent potential alternative or adjunct antimycobacterial agents capable of targeting virulence-associated mechanisms. Furthermore, phytochemical profiling and bioactivity-guided analysis of medicinal plants are necessary for identifying bioactive lead compounds, understanding mechanisms of action, and scientifically validating the traditional use of medicinal plants in the management of tuberculosis and other mycobacterial infections. The aim of this study was to evaluate the phytochemical composition, antioxidant, anti-inflammatory, and antimycobacterial activities of *D. rotundifolia* and *L. javanica*, and to isolate and characterize active compounds using bioassay-guided fractionation. By identifying fatty acids with antimycobacterial activity, this work provides potential scaffolds for the development of novel therapeutics against TB and related infections.

## 2. Results

### 2.1. Extraction Yield of Crude Extracts

**Figure 1:**
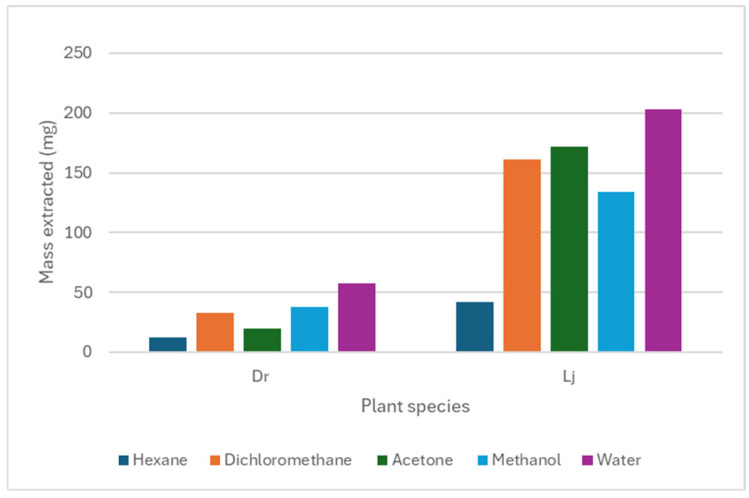
Mass of *Dombeya rotundifolia* (Dr) and *Lippia javanica* (Lj) extracts, extracted using different solvents with varying polarity.

The most polar solvent, water, proved to be the most effective for extracting compounds, yielding the highest masses. The water extract of *L. javanica* exhibited the highest yield at 203 mg, followed by the water extract of *D. rotundifolia* at 57.6 mg. Conversely, the lowest yields were obtained with hexane, at 12.4 mg for *D. rotundifolia* and 42 mg for *L. javanica* (Figure 1).

### 2.2. Quantification of Polyphenolic and Antioxidant Activity

*D. rotundifolia* exhibited higher levels of total phenolics and flavonoids than *L. javanica*, with the hexane extract containing the highest concentrations of total phenolics (186.29 ± 0.70 mg GAE/g) and flavonoids (122.91 ± 2.82 mg QE/g) (Table 1). In contrast, the acetone extract of *L. javanica* contained lower levels of phenolics (5.43 ± 0.24 mg GAE/g) and flavonoids (1.19 ± 0.11 mg QE/g). Consistent with its higher polyphenolic content, *D. rotundifolia* demonstrated strong antioxidant activity in the DPPH assay, with an EC_50_ value of 51.84 μg/mL, which was comparable to that of the positive control, ascorbic acid (EC_50_ = 33.31 μg/mL). In the ferric-reducing power assay, *L. javanica* exhibited moderate antioxidant capacity with an EC_50_ value of 270.1 μg/mL, which was less potent than ascorbic acid (EC_50_ = 91.70 μg/mL) (Table 1).

### 2.3. Anti-Inflammatory Activity

**Figure 2:**
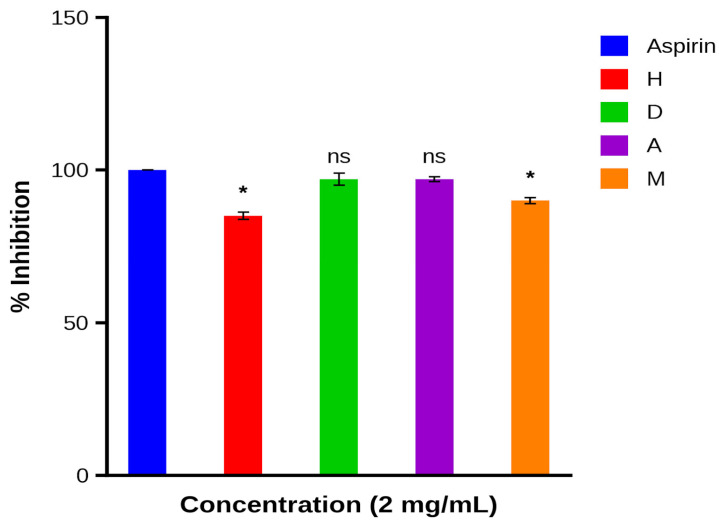
Inhibition of heat-induced albumin denaturation by *Dombeya rotundifolia* extracts. Data are presented as the mean ± standard deviation of the triplicate experiments. One-way ANOVA followed by Dunnett’s multiple comparisons tests was used to compare the plant extracts with standard control Aspirin. Significance was detected where *p* < 0.05. ns: not significant. H—Hexane, D—Dichloromethane, A—Acetone, M—Methanol, ns = non-significant difference (aspirin and plant extracts), (*) = significant difference (between aspirin and plant extracts).

At 2 mg/mL, the inhibition of protein denaturation by the plant extracts ranged from 85% to 97% (Figure 2). The dichloromethane and acetone extracts of *D. rotundifolia* exhibited the highest activity, with inhibition rates of 97.54% and 97.10%, respectively (Figure 2).

### 2.4. Antimycobacterial Activity

For *D. rotundifolia*, the acetone and dichloromethane extracts exhibited the strongest antimycobacterial activity, with MIC values of 0.16 mg/mL, while the water extract was the least active (MIC = 0.42 mg/mL). In *L. javanica*, the dichloromethane extract showed moderate activity with an MIC of 0.63 mg/mL, whereas the methanol and water extracts were largely inactive, with MIC values greater than 2.5 mg/mL (Table 2).

### 2.5. Isolation and Characterization of Antimycobacterial Compounds

**Figure 3:**
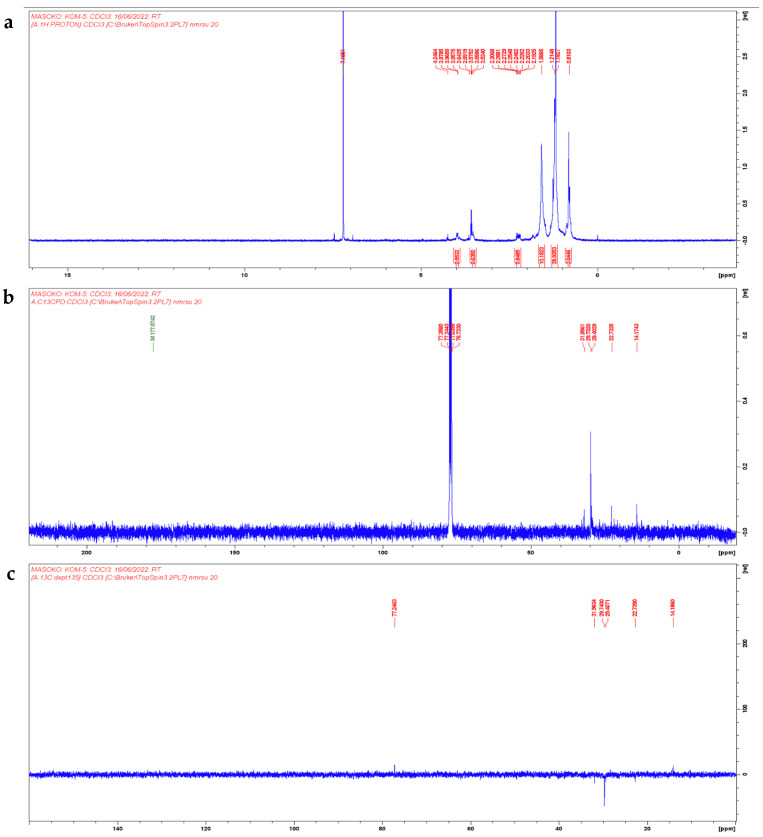
NMR spectra of isolated fraction 1 showing (**a**) ^1^H NMR spectrum, (**b**) the proton-decoupled ^13^C CPD NMR spectrum, and (**c**) the ^13^C DEPT-135 NMR spectrum. The red numbers represent peak-picking labels automatically generated by the NMR software. They indicate the chemical shift values (δ, ppm) of peaks that the software has detected.

**Figure 4:**
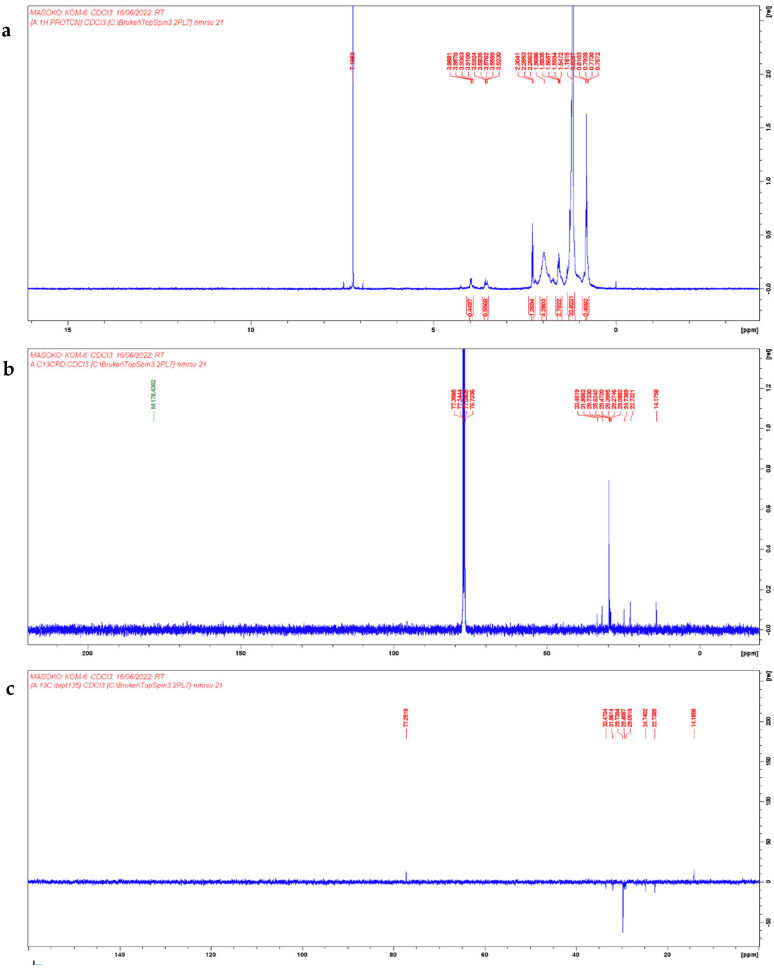
NMR spectra of isolated fraction 2 (**a**) ^1^H NMR spectrum, (**b**) ^13^C CPD NMR spectrum, and (**c**) ^13^C DEPT-135 NMR spectrum. The red numbers represent peak-picking labels automatically generated by the NMR software. They indicate the chemical shift values (δ, ppm) of peaks that the software has detected.

**Figure 5:**
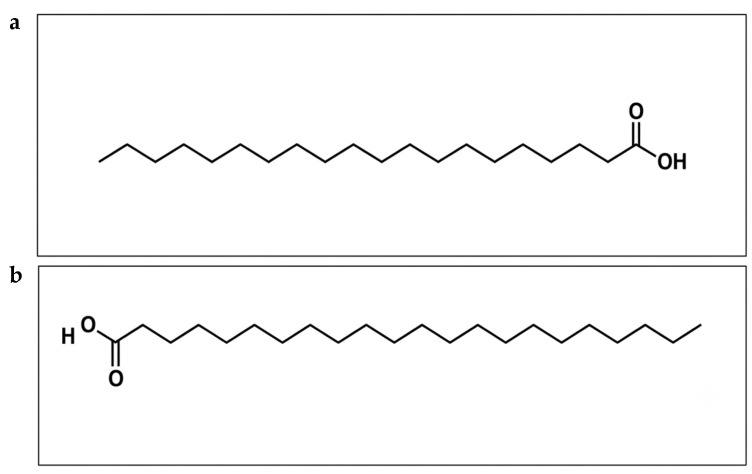
Structure of fraction 1 (Eicosanoic acid) (**a**) and Structure of fraction 2 (Docosanoic acid (Behenic acid) (**b**) from the leaves of *Dombeya rotundifolia*.

The crude extracts obtained through serial exhaustive extraction showed that the dichloromethane (D1–D3) and acetone (A1–A3) fractions exhibited lower MIC values (0.26–0.42 mg/mL) than the hexane fractions (MIC ≥ 0.52 mg/mL) and methanol fractions (MIC > 2.5 mg/mL) (Table 3). Based on their notable antimycobacterial activity, the dichloromethane and acetone extracts were combined and subjected to further fractionation. The isolated fractions were characterized using NMR spectroscopy (Figure 3 and Figure 4). Table 4 and Table 5 present the ^1^H and ^13^C NMR chemical shift data of the isolated compounds in comparison with literature values. Analysis of the spectra enabled the tentative characterization of the two active fractions as eicosanoic acid (arachidic acid) and docosanoic acid (behenic acid) (Figure 5).

### 2.6. Cytotoxicity of Crude Extracts

**Figure 6:**
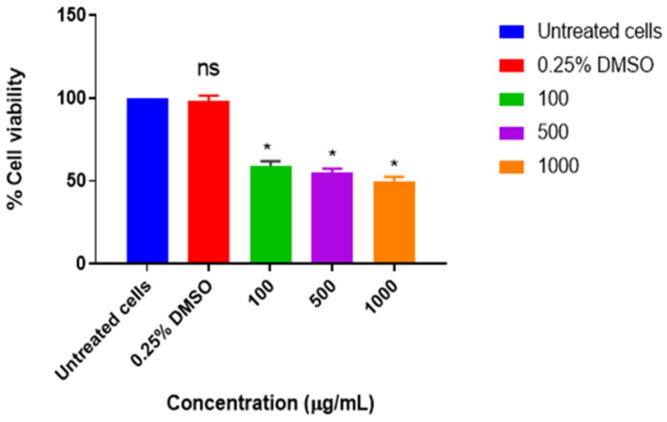
Cytotoxic effect of *Dombeya rotundifolia* (acetone extract) against THP-1 macrophages. Data is presented as the mean ± standard deviation of duplicate experiments. One-way ANOVA coupled with Dunnett’s multiple comparisons test was used. Significant difference was observed when (*): *p* < 0.05. ns: not significant.

A concentration-dependent decrease in cell viability was observed (Figure 6). At 100 µg/mL, the extract maintained 59% cell viability, which declined to 49% at 1000 µg/mL. The half-maximal lethal concentration (LC_50_) was determined to be 286.31 µg/mL based on a dose–response curve.

## 3. Discussion

Plant-derived medications are a significant source of bioactive compounds and continue to play a critical role in the development of new drugs [22]. The extraction procedure separates these active compounds from inert plant material using a suitable solvent and standard extraction procedures [18]. Water was the most efficient extractant for both plants due to its high polarity, which facilitates the extraction of a wide range of polar compounds (Figure 1). The water extract of *L. javanica* yielded the highest mass at 203 mg, followed by the water extract of *D. rotundifolia* at 57.6 mg. Conversely, the non-polar hexane extracts yielded the lowest masses, with *D. rotundifolia* at 12.4 mg and *L. javanica* at 42 mg (Figure 2). This aligns with the understanding that hexane primarily extracts non-polar compounds [23].

*D. rotundifolia* contained notable levels of phenolics and flavonoids in all the plant extracts, especially in the hexane extract. While hexane is a highly non-polar solvent traditionally used to isolate lipids and waxes, the unexpected detection of high total phenolic content (TPC) and total flavonoid content (TFC) in the hexane extract can be attributed to distinct chemical and methodological factors. Structurally, certain plant species synthesize hydrophobic, low-polarity phenolics, such as highly methoxylated flavonoids, alkylated phenols, or phenolic lipids, which preferentially partition into non-polar solvents rather than hydrophilic phases [24]. Furthermore, hexane aggressively extracts lipophilic constituents, including fats, waxes, essential oils, carotenoids, chlorophyll derivatives, and tocopherols, which may facilitate the co-extraction and solubilization of moderately hydrophobic phenolic compounds. However, the observed TPC and TFC values should be interpreted with caution due to the limitations of the colorimetric assays employed. The commonly used Folin–Ciocalteu assay relies on a non-specific single-electron transfer redox reaction and therefore measures the overall reducing capacity of an extract rather than phenolic compounds exclusively [25]. Similarly, the aluminum chloride assay used for TFC determination may be influenced by non-flavonoid constituents capable of forming complexes or interfering with the assay response. Consequently, lipophilic non-phenolic antioxidants co-extracted by hexane, including carotenoids, chlorophyll derivatives, tocopherols, and unsaturated fatty acids, may contribute to the measured TPC and TFC values, potentially resulting in an overestimation of the actual phenolic and flavonoid contents [25,26]. These compounds may also contribute to the antioxidant activity observed in the hexane extract. Therefore, chromatographic techniques such as HPLC-DAD or LC-MS are required to distinguish genuine lipophilic phenolics and flavonoids from matrix-induced false positives [25]. Researchers have reported the polyphenolic content of *D. rotundifolia* in crude acetone extract. The total phenolic content ranges from 45.3 to 259.0 mg GAE/g, and flavonoid content ranges from 19.90 to 35.4 mg QE/g [11]. These findings for phenolics are consistent with the reported results; however, the flavonoid content was higher (53.07 mg QE/g) than the reported range (19.90 to 35.4 mg QE/g). Such variations in phytochemical composition are common and can be attributed to environmental factors such as soil composition, seasonal variations, and temperature, as well as intrinsic biological factors, including the plant’s developmental stage [27]. In contrast, *L. javanica* exhibited significantly lower levels of phenolics and flavonoids, which aligns with previous reports [28]. The high phenolic content in *D. rotundifolia* suggests its potential to scavenge reactive free radicals and contribute to human health, as flavonoids and phenols are known to possess strong antioxidant activity [29].

The antioxidant activity of the plant extracts is expressed as the EC_50_ value, which represents the concentration required to scavenge 50% of free radicals (Table 2). In the DPPH assay, *D. rotundifolia* extracts showed high antioxidant potential with an EC_50_ value of 51.84 μg/mL, which is close to the positive control, ascorbic acid (EC_50_ = 33.31 μg/mL). This strong activity is likely due to the high phenolic content observed in this plant, as phenolic compounds are well known for their antioxidant and free radical scavenging abilities [30]. The ferric-reducing power assay, which measures the electron-donating capacity of the extracts, showed that *L. javanica* exhibited a moderate ability to reduce ferric ions with an EC_50_ of 270.70 μg/mL. While this indicates antioxidant potential, it is higher compared to the positive control (ascorbic acid, EC_50_ = 91.70 μg/mL). The overall antioxidant activity observed in both plants may be attributed, at least in part, to the presence of flavonoids and phenolic compounds, which are known for their redox properties and their potential role in mitigating oxidative stress-related disorders [31]. However, it should be noted that other antioxidant phytochemicals, including non-phenolic constituents may also contribute to the observed activity, particularly in non-polar extracts. Therefore, the antioxidant activity detected in this study likely reflects the combined effects of multiple bioactive constituents rather than phenolics and flavonoids alone. Antioxidant phytochemicals are considered important contributors to the therapeutic potential of plant-derived products against diseases such as tuberculosis [32].

Maintaining body temperature within 36–37 °C is essential for homeostasis, as deviations can cause protein unfolding or aggregation, impairing function [33]. During bacterial infection, the host mounts a pro-inflammatory response that induces fever. Febrile-range temperatures (≈38–41 °C) enhance leukocyte trafficking, phagocytosis, and lymphocyte activation, thereby supporting pathogen clearance [34]. The anti-inflammatory activity was assessed using the egg albumin protein denaturation assay with aspirin as a positive control. The results (Figure 2) demonstrated that inhibition of protein denaturation ranged from 85% to 97% for the plant extracts at a concentration of 2 mg/mL, with the dichloromethane and acetone extracts of *D. rotundifolia* showing the highest activity (97.54% and 97.1%, respectively). Lower concentrations of these extracts exhibited undetectable activity, suggesting that the anti-inflammatory effect is dose-dependent and requires higher concentrations. The activity of the dichloromethane and acetone extracts was statistically non-significant when compared to aspirin, which aligns with their potent anti-inflammatory properties and supports previous findings [10,35].

The antimycobacterial activity of the plant extracts was evaluated in terms of minimum inhibitory concentration (MIC), minimum bactericidal concentration (MBC), and total activity (Table 3). MIC values are considered strong when <0.1 mg/mL, moderate between 0.1 and 0.625 mg/mL, and weak when >0.625 mg/mL [36]. For *D. rotundifolia*, the acetone and dichloromethane extracts exhibited moderate activity with MIC values of 0.16 mg/mL, while the water extract was the least active with an MIC of 0.42 mg/mL. Although these results indicate measurable antimycobacterial activity, it is important to note that the observed effects against *M. smegmatis* should be interpreted as preliminary, since *M. smegmatis* is a non-pathogenic surrogate model and differs significantly from *Mycobacterium tuberculosis* in terms of cell wall composition, virulence, and drug susceptibility. Therefore, while useful for initial screening, this model does not fully predict activity against the pathogenic organism.

The acetone and dichloromethane extracts of *D. rotundifolia* were previously reported to show activity against *Escherichia coli* with MIC values of 0.32 mg/mL [37]. Additionally, the leaf acetone extract had moderate activity against *Bacillus cereus*, *Enterococcus faecalis*, and *Pseudomonas aeruginosa* with MIC values of 0.39 mg/mL [38]. Compounds isolated from *D. rotundifolia*, such as lauric acid and myristic acid, have been reported to be effective against *Mycobacterium* species [17,18]. To our knowledge, this appears to be the first report on the antimycobacterial activity of *D. rotundifolia* against *M. smegmatis*.

Conversely, the dichloromethane extract of *L. javanica* had a moderate MIC of 0.63 mg/mL, while the water and methanol extracts were largely inactive (MIC > 2.5 mg/mL). There are reports on the antimycobacterial activity of *L. javanica* against *Mycobacterium* species [15,16], which support the findings from this study. The higher activity of the dichloromethane extract in both plants can be attributed to its ability to extract lipophilic compounds such as alkaloids, terpenoids, and fatty acids, which are known to possess antibacterial effects. All extracts showed an MBC value greater than 2.5 mg/mL, suggesting that the extracts are bacteriostatic rather than bactericidal, which is an important consideration for their potential therapeutic application. The total activity, which indicates the extent to which a gram of plant material can be diluted and still inhibit bacterial growth, was highest in the dichloromethane extracts of both plants, with *D. rotundifolia* having a total activity of 203.13 mg/L.

The MTT cytotoxicity assay (Figure 6) showed that the acetone extract of *D. rotundifolia* reduced cell viability in THP-1 macrophages, particularly at higher concentrations (59% viability at 100 µg/mL and 49% at 1000 µg/mL). The LC_50_ value of 286.31 µg/mL indicates moderate cytotoxicity toward mammalian cells. The selectivity index (SI = LC_50_/MIC) was calculated to be 1.79, indicating that cytotoxic and antimycobacterial effects occur within a relatively close concentration range. Although an SI value greater than 1 suggests some degree of selectivity toward bacterial cells [39], the relatively low margin highlights limited therapeutic selectivity of the crude extract. This suggests that, in its current crude form, the extract may not be directly suitable for therapeutic application without further fractionation or optimization to improve safety and reduce cytotoxic constituents. While some studies using preliminary assays such as the brine shrimp lethality test have suggested low toxicity [40], the results from the MTT assay on a human-derived macrophage cell line provide a more clinically relevant indication of cytotoxic potential. Therefore, careful dose optimization and further safety profiling will be required in future studies to determine a therapeutically acceptable window.

Bioassay-guided fractionation was employed to isolate active antimycobacterial fatty acid fractions from *D. rotundifolia* (Table 4 and Table 5). The two fractions showed moderate antimycobacterial activity with a collective MIC of 0.25 mg/mL (Table 3). This MIC value is higher (less potent) than that of the crude dichloromethane and acetone extracts (0.16 mg/mL), suggesting that the activity of the crude extracts may be due to a synergistic effect between multiple compounds.

Other fatty acids have previously been isolated from *D. rotundifolia* (ethanolic leaf extracts), such as lauric, myristic, stearic, and palmitic acids [41]. It has been reported that lupeol and β-sitosterol have been isolated from the stem bark [42]. Fatty acids have a well-documented history of antimicrobial properties, and their mechanisms of action often involve disruption of bacterial cell membranes [43]. Our findings align with previous studies that have also reported the antimycobacterial activity of fatty acids such as myristic and lauric acid [44]. Furthermore, other studies have shown that eicosanoic and docosanoic acids from the methanolic extract of the root of *Paullinia pinnata* L exhibit antibacterial activity against various bacterial species, supporting their potential as therapeutic agents [45,46]. The findings from this study, combined with evidence of host-mediated production of these fatty acids during bacterial infection [47], underscore their potential role as therapeutic agents. An interesting finding of this study was the identification of fatty acid-rich fractions as the major contributors to the antimycobacterial activity of *D. rotundifolia*. Unlike many bioactive secondary metabolites that occur in low concentrations and require extensive purification, fatty acids can be enriched using relatively simple and cost-effective approaches such as solvent partitioning and defatting procedures. This may facilitate the development of standardized extracts with consistent phytochemical profiles and biological activity.

## 4. Materials and Methods

### 4.1. Chemicals and Reagents

The following analytical-grade chemicals and reagents were used: hexane, dichloromethane, acetone, methanol, Folin–Ciocalteu reagent, sodium carbonate, aluminum chloride, L-ascorbic acid, sodium nitrite, sodium acetate, quercetin, gallic acid, 2,2-diphenyl-1-picrylhydrazyl (DPPH), potassium ferricyanide, trichloroacetic acid, ferric chloride, middlebrook 7H9 broth, and p-iodonitrotetrazolium chloride (all from Merck, Darmstadt, Germany); glycerol and oleic albumin dextrose catalase (OADC) supplement (Fluka, Buchs, Switzerland), fetal bovine serum (FBS) (Merck, Modderfontein, South Africa), phorbol-12-myristate-13-acetate (PMA) (Sigma Aldrich^®^, St. Louis, MO, USA), 3-(4,5-dimethylthiazol-2-yl)-2,5-diphenyl tetrazolium bromide (MTT) (Merck, Modderfontein, South Africa).

### 4.2. Plant Material and Extraction Procedure

Leaves of *D. rotundifolia* (voucher UNIN 12296) and *L. javanica* (voucher UNIN 1220265) were collected from the University of Limpopo Botanical Garden and Lowveld National Botanical Garden (Mpumalanga, South Africa). The plants were identified by Dr. Egan Bronwyn and deposited at the Larry Leach Herbarium (University of Limpopo). Leaves were dried at ambient temperature in the dark for two weeks, powdered, and stored in airtight containers.

For preliminary screening, 1 g of powdered material was extracted with 10 mL of solvents (H—hexane, D—dichloromethane, A—acetone, M—methanol, and W—water) separately in 50 mL centrifuge tubes to obtain a crude extract for each solvent. The mixtures were shaken separately for 10 min at a speed of 200 rpm in a shaking incubator (New Brunswick Scientific Co., INC, Edison, NJ, USA). Extracts were filtered, evaporated, weighed, and reconstituted in acetone at 10 mg/mL. Water extracts were reconstituted in distilled water. The quantity of the plant extracts was determined by subtracting the mass of the empty pre-weighed glass vials from the mass of the dried crude extracts in the vials.

### 4.3. Phytochemical Quantification

#### 4.3.1. Total Phenolic Content

Total phenolics were quantified using the Folin–Ciocalteu method by Tambe and Bhamber [48]. The plant extracts included *D. rotundifolia* (hexane, dichloromethane, acetone, methanol, and water extracts) and *L. javanica* (acetone extract). Plant extracts (1 µg/mL) were reacted with 250 µL Folin–Ciocalteu reagent and 1.25 mL of 7% sodium carbonate, then incubated for 30 min in the dark at room temperature. Absorbance was read at 725 nm using the ultraviolet/visible (UV/VIS) spectrophotometer (Genesys 10S UV-VIS, Menlo Park, CA, USA). Gallic acid was used as a standard (1.25–0.08 mg/mL), and results were expressed as mg gallic acid equivalents per gram extract (mg GAE/g) using the equation: y = 3.025x − 0.1141. All assays were performed in triplicate.

#### 4.3.2. Total Flavonoid Content

Flavonoid content was assessed using the aluminum chloride colorimetric assay by Tambe and Bhamber [48]. The plant extracts: for *D. rotundifolia* (hexane, dichloromethane, acetone, methanol, and water, *L. javanica* (acetone extract). Extracts (10 mg/mL) were reacted with sodium nitrite, aluminum chloride, and sodium hydroxide, and brought to 10 mL volume. After incubation, absorbance was recorded at 510 nm. Quercetin standards (500–31.25 µg/mL) were used (y = 0.6522x − 0.0006), and results expressed as mg quercetin equivalents per gram extract (mg QE/g). All assays were conducted in triplicate.

### 4.4. Quantitative Antioxidant Activity

#### 4.4.1. DPPH Radical Scavenging Assay

The antioxidant activity of acetone extracts was evaluated using the DPPH assay by Chigayo et al. [29]. Extracts and L-ascorbic acid (standard) were prepared in concentrations ranging from 250 to 15.63 µg/mL. Each 1 mL sample was mixed with 2 mL of 0.2 mmol/L DPPH solution in methanol, vortexed, and incubated in the dark for 30 min. Absorbance was measured at 517 nm using the ultraviolet/visible (UV/VIS) spectrophotometer (Genesys 10S UV-VIS, Menlo Park, CA, USA). Percentage inhibition was calculated as:

$$\%Inhibition=\frac{Ac-As}{Ac}\times 100$$

where Ac is the absorbance of the control solution and As is the absorbance of the plant extract.

#### 4.4.2. Ferric Reducing Power Assay

Reducing power was assessed using the method of Vijayalakshmi and Ruckmani [49]. Acetone extracts and ascorbic acid (standard) were serially diluted (625–39 µg/mL). Each sample (2.5 mL) was mixed with 2.5 mL sodium phosphate buffer (0.2 M, pH 6.6) and 2.5 mL of 1% potassium ferricyanide. After incubation at 50 °C for 20 min, 2 mL of 10% trichloroacetic acid was added. A 5 mL aliquot of the supernatant was combined with 5 mL of distilled water and 1 mL of 0.1% ferric chloride. Absorbance was measured at 700 nm. Blank samples used acetone instead of the extract. All assays were performed in triplicate.

### 4.5. Anti-Inflammatory Activity (Protein Denaturation Assay)

The inhibition of protein denaturation was evaluated using egg albumin denaturation assay by Uttra and Rahman et al. [50,51]. The plant extracts: *D. rotundifolia* (hexane, dichloromethane, acetone and methanol). The reaction mixture (5 mL) contained 0.2 mL of fresh egg albumin, 2.8 mL of 0.05 M phosphate buffer (pH 6.6), and 2 mL of plant extracts or aspirin at concentrations of 2, 1, and 0.5 mg/mL. A product control was prepared using 3 mL buffer and 2 mL extract to account for extract colour, while the positive control contained 0.2 mL albumin and 4.8 mL buffer. Samples were incubated at 37 ± 2 °C for 15 min, then heated at 70 °C for 5 min. After cooling to room temperature for 30 min, absorbance was measured at 660 nm using the ultraviolet/visible (UV/VIS) spectrophotometer (Genesys 10S UV-VIS, Menlo Park, CA, USA). Phosphate buffer served as the blank. All experiments were conducted in triplicate. The percentage inhibition of protein denaturation was calculated as:

$$\%Anti-denaturation=\frac{Ac-As}{Ac}\times 100$$

where, Ac is the absorbance of the control solution and As is the absorbance of the plant extract.

### 4.6. Antimycobacterial Activity Screening

#### 4.6.1. Microorganism and Culture Conditions

*Mycobacterium smegmatis* was kindly provided by Prof. Green (Department of Biotechnology and Food Technology, University of Johannesburg, Gauteng, South Africa). The strain was maintained on Middlebrook 7H9 agar supplemented with glycerol and Middlebrook oleic albumin dextrose catalase (OADC) growth supplement. For experimental use, the mycobacterium was cultured in Middlebrook 7H9 broth containing glycerol and OADC and incubated at 37 °C for 24 h.

#### 4.6.2. Broth Microdilution Assay

Minimum inhibitory concentrations (MICs) (2.5–0.019 mg/mL) were determined using the microplate broth dilution method by Eloff [52]. The plant extracts for *D. rotundifolia* and *L. javanica* (hexane, dichloromethane, acetone, methanol, and water) Plant extracts were dissolved in acetone to a final concentration of 10 mg/mL. Acetone was used as solvent for reconstituting due to its safety profile and very low toxicity to assay organisms. In a 96-well microtiter plate, 100 µL of sterile distilled water was added to each well. Then, 100 µL of extract was added to the first well and serially diluted 2-fold across the plate. A 100 µL aliquot of *M. smegmatis* culture was added to each well. Rifampicin served as positive control and acetone as the negative control. Plates were incubated at 37 °C for 24 h. After incubation, 40 µL of 0.2 mg/mL p-iodonitrotetrazolium violet (INT) was added to each well, and plates were incubated for an additional 30 min. A reduction in purple colour indicated bacterial growth. The MIC was defined as the lowest concentration of extract that prevented colour change. All assays were performed in triplicate. The total activity (mL/g) was calculated by dividing the mass (mg) of the extract obtained from 1 g of plant material by its MIC value (mg/mL).

### 4.7. Cell Viability Assay

The 3-(4,5-dimethylthiazol-2-yl)-2,5-diphenyl tetrazolium bromide (MTT) assay by Mosmann [53] was used to assess cytotoxicity against THP-1 cells. Cells were cultured in RPMI 1640 medium supplemented with 10% Fetal Bovine Serum (FBS) and seeded at 2 × 10^5^ cells/well in 96-well plates. Differentiation was induced using 50 ng/mL Phorbol 12-myristate 13-acetate (PMA) for 48 h, followed by a 24 h recovery in fresh media [54,55]. Acetone extracts (1000, 500, 100 µg/mL) were prepared in DMSO (final 0.25%) and added to the wells for 24 h. After treatment, 20 µL of 0.5 mg/mL MTT in PBS was added and incubated for 4 h. Medium was removed, and 100 µL DMSO was added to dissolve the formazan crystals. Absorbance was measured at 540 nm (Promega microplate reader). The assays were performed in duplicate. Cell viability was expressed relative to untreated controls.

### 4.8. Bioassay-Guided Fractionation of Antimycobacterial Compounds

**Figure 7:**
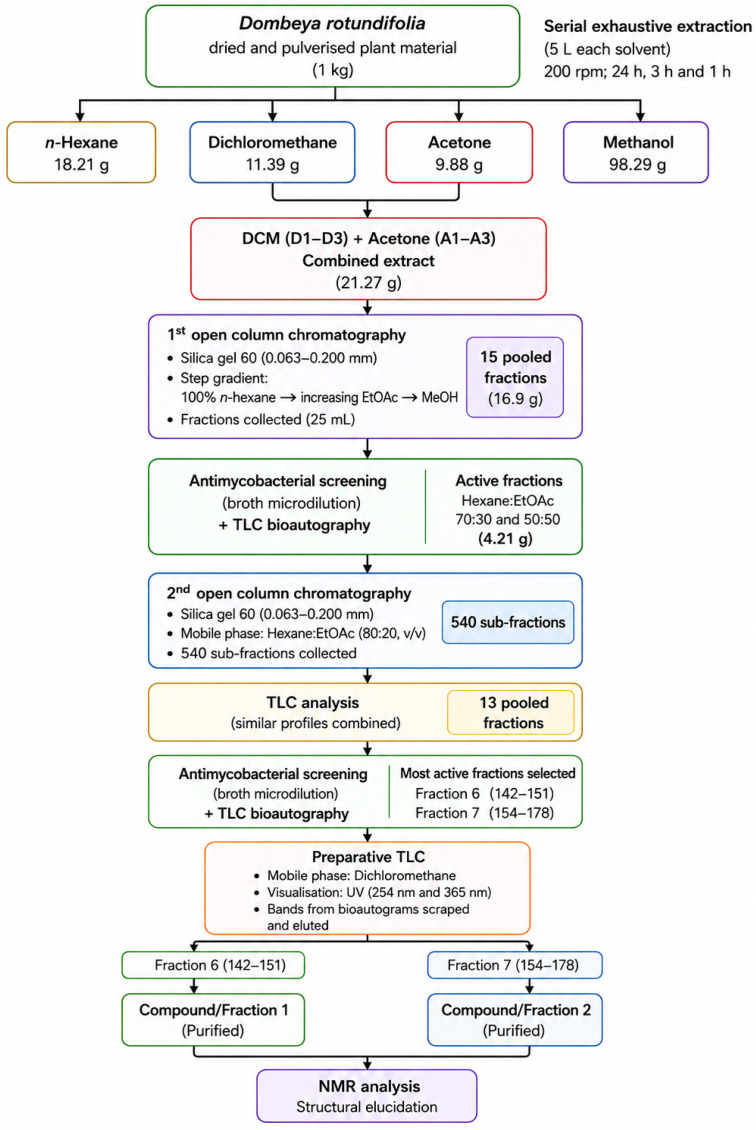
Bioassay-guided fractionation and isolation scheme for compounds from *Dombeya rotundifolia* leaves. It outlines the progression from crude plant material through sequential solvent extraction, chromatographic separation, fraction pooling, and purification, culminating in the isolation of compounds for structural elucidation by NMR spectroscopy.

Bioassay-guided fractionation was performed to isolate antimycobacterial compounds from *Dombeya rotundifolia*. Briefly, dried and pulverized plant material (1 kg) was subjected to serial exhaustive extraction using solvents of increasing polarity. The plant material was extracted with 5 L of n-hexane and agitated overnight at 200 rpm. The extract was filtered and concentrated under reduced pressure using a rotary evaporator (Büchi R-114) (Marshall Scientific, Hampton, VA, USA). The extraction process was repeated twice on the same plant material for 3 h and 1 h, respectively, to ensure exhaustive recovery of constituents. The residual plant material was subsequently extracted sequentially with 5 L dichloromethane, acetone, and methanol following the same procedure (Figure 7). All extracts were concentrated to dryness under a stream of air at room temperature and weighed. The extraction yielded 18.21 g, 11.39 g, 9.88 g, and 98.29 g of hexane, dichloromethane, acetone, and methanol extracts, respectively.

Based on preliminary antimycobacterial screening, the dichloromethane (D1–D3) and acetone (A1–A3) extracts exhibited the highest activity and were therefore combined for further purification. A total of 21.27 g of the combined extract was subjected to open-column chromatography using a glass column (47 cm × 3 cm) packed with silica gel 60 (0.063–0.200 mm particle size). Elution was performed using a stepwise gradient system beginning with 100% n-hexane, followed by increasing proportions of ethyl acetate and finally methanol to increase solvent polarity [56]. Fractions of 25 mL were collected and monitored by thin-layer chromatography (TLC). Fractions displaying similar TLC profiles were combined to yield 15 pooled fractions (16.9 g). The pooled fractions were screened for antimycobacterial activity using the broth microdilution assay and analyzed by TLC bioautography to identify chromatographic bands associated with antimycobacterial activity. Based on the bioassay and bioautography results, the most active fractions, corresponding to those eluted with hexane:ethyl acetate (70:30 and 50:50), were selected for further purification. A total of 4.21 g of these active fractions was subjected to a second open-column chromatography (60 cm × 2 cm) packed with silica gel and eluted using hexane: ethyl acetate (80:20, *v*/*v*) as the mobile phase. A total of 540 sub-fractions were collected and analyzed by TLC on silica gel 60 F254 plates (20 × 20 cm, 0.25 mm thickness). Sub-fractions exhibiting similar TLC profiles were pooled to yield 13 combined fractions.

The 13 pooled fractions were further evaluated using antimycobacterial assays and TLC bioautography to track the active constituents. Fraction 6 (sub-fractions 142–151) and Fraction 7 (sub-fractions 154–178) exhibited the strongest antimycobacterial activity and contained distinct bioactive bands on TLC bioautograms. These fractions were therefore selected for further purification by preparative TLC. Preparative TLC plates were developed using dichloromethane as the mobile phase and visualized under ultraviolet light at 254 and 365 nm. The bands corresponding to the bioactive regions identified by bioautography were carefully scraped from the silica gel and eluted with a suitable solvent. Purification of Fraction 6 yielded Fraction 1, while purification of Fraction 7 yielded Fraction 2. The two purified fractions were concentrated under reduced pressure and subsequently characterized by nuclear magnetic resonance (NMR) spectroscopy for structural elucidation which tentatively exhibited the presence of a fatty acids type of metabolite.

### 4.9. Structural Elucidation of Isolated Fractions

The active fractions were sent to the Department of Chemistry at the University of Limpopo for structural identification using NMR techniques. Then fractions were analyzed using 1-dimensional NMR (^1^H, ^13^C and DEPT 135). About 5 mg of a sample (fraction 1 and fraction 2 separately) was dissolved in chloroform and run using 400 MHz NMR Spectrometer (Bruker) (Billerica, MA, USA) at 400 MHz. Dr. Ofentse Mazimba, from Botswana International University of Science and Technology, Department of Chemical and Forensic Sciences, assisted with the analysis of the NMR spectra and structure elucidation of the fractions.

### 4.10. Statistical Analysis

All experiments were conducted in triplicate, and results were expressed as mean ± standard deviation (SD). Statistical significance was determined using one-way ANOVA followed by Dunnett’s multiple comparisons test (GraphPad Prism version 9.0). Differences were considered statistically significant at *p* < 0.05.

## 5. Conclusions

This study demonstrated that *D. rotundifolia* and *L. javanica* contain diverse phytochemicals with antioxidant, anti-inflammatory, and antimycobacterial activities. The acetone and dichloromethane fractions of *D. rotundifolia* exhibited the most notable antimycobacterial activity against *M. smegmatis*, although the observed effects should be regarded as moderate and preliminary due to the limitations of this surrogate model in predicting activity against *M. tuberculosis*. Bioassay-guided fractionation further led to the isolation of two fatty acid fractions with moderate activity, suggesting that these common and chemically accessible metabolites. The findings support the ethnomedicinal use of *D. rotundifolia* in respiratory ailments, including tuberculosis; however, the relatively close proximity between cytotoxic and antimycobacterial concentrations indicates limited selectivity of the crude extract and highlights the need for careful toxicological profiling. Nonetheless, the identification of fatty acids as active constituents presents a practical advantage, as these compounds can be enriched using relatively simple and cost-effective extraction approaches, supporting the feasibility of developing standardized extract-based preparations. Further studies against *M. tuberculosis*, together with mechanistic investigations are required to confirm the therapeutic relevance and safety of these compounds and to assess their potential as phytopharmaceutical ingredients or lead molecules for anti-TB drug development. The bioactive extracts and fractions will in future be subjected to liquid chromatography-mass spectrometry (LC-MS) and gas chromatography-mass spectrometry (GC-MS) analysis to determine their phytochemical compositions.

## Figures and Tables

**Table 1:** Total polyphenolic contents and antioxidant activity of the acetone crude extracts.

Plant/Control	Extracts	TPC (mg GAE/g Extract)	TFC (mg QE/g Extract)	Free-Radical Scavenging Activity (DPPH)		Ferric Reducing Power	
				EC_50_ (µg/mL)	R^2^	EC_50_ (µg/mL)	R^2^
*D. rotundifolia*	H	186.29 ± 0.70 ^f^	122.91 ± 2.82 ^e^	51.84 ^b^	0.91	356.2 ^c^	0.99
	D	132.67 ± 0.70 ^e^	65.87 ± 0.87 ^d^				
	A	49.03 ± 0.70 ^b^	53.07 ± 1.19 ^c^				
	M	54.72 ± 1.82 ^c^	28.23 ± 1.19 ^b^				
	W	99.48 ± 1.64 ^d^	55.29 ± 1.95 ^c^				
*L. javanica*	A	5.43 ± 0.24 ^a^	1.19 ± 0.11 ^a^	98.82 ^c^	0.97	270.1 ^b^	0.91
Ascorbic acid		-	-	33.31 ^a^	0.97	91.70 ^a^	0.99

The results are presented as the mean ± standard deviation of triplicate determinations. For TPC and TFC, differences among the extracts were analyzed using one-way ANOVA followed by Tukey’s multiple comparison post hoc test. Significance was accepted at *p* < 0.05, and different letters within a column indicate significant differences. For antioxidant assays, one-way ANOVA followed by Dunnett’s multiple comparison test was used to compare the acetone extracts with the standard control (ascorbic acid). Statistical significance was observed at *p* < 0.0001. H = hexane; D = dichloromethane; A = acetone; M = methanol; W = water; GAE = gallic acid equivalents; QE = quercetin equivalents; TPC = total phenolic content; TFC = total flavonoid content.

**Table 2:** Antimycobacterial activity of plant extracts against *M. smegmatis*.

Plants	H			D			A			M			W		
	MIC	MBC	TA	MIC	MBC	TA	MIC	MBC	TA	MIC	MBC	TA	MIC	MBC	TA
*D. rotundifolia*	0.21	>2.5	59.05	0.16	>2.5	203.13	0.16	>2.5	123.75	0.26	>2.5	146.54	0.42	>2.5	137.14
*L. javanica*	2.5	˃2.5	16.80	0.63	>2.5	255.56	2.5	>2.5	68.80	>2.5	>2.5	-	>2.5	>2.5	-

H—Hexane, D—Dichloromethane, A—Acetone, M—Methanol, W—Water, MIC = Minimum Inhibitory Concentration, MBC = Minimum Bactericidal Concentration, TA = Total antimycobacterial activity, units for MIC and MBC-mg/mL, units for Total antimycobacterial activity = mL/g, Rifampicin (positive control and acetone (negative control)).

**Table 3:** Antimycobacterial activity of fractions against *M. smegmatis*.

Serial exhaustive extraction fractions															
*D. rotundifolia*	H1	H2	H3	D1	D2	D3	A1	A2	A3	M1	M2	M3	-	-	-
	MIC	MIC	MIC	MIC	MIC	MIC	MIC	MIC	MIC	MIC	MIC	MIC			
	˃2.5	˃2.5	0.52	0.26	0.26	0.42	0.21	0.42	0.42	˃2.5	˃2.5	˃2.5	-	-	-
Isolated Fractions															
Fraction 1	0.25														
Fraction 2	0.25														
Rifampicin	0.04														

H (1–3)—Hexane, D (1–3)—Dichloromethane, A (1–3)—Acetone, M (1–3)—Methanol, MIC = Minimum Inhibitory Concentration, units for MIC—mg/mL.

**Table 4:** ^1^H (300 MHz) and ^13^C (75.4 MHz) data of fraction 1.

Position	δH	δC	δH	δC
	Literature ^a^		Isolated fraction	
1		179.4 (s)		177.5
2	2.28, 2H, t, J = 7.8 Hz	33.9 (t)	1.58, 2H, brs	31.9
3–19	1.19, 36H, brs	22.7, 29.1–29.7 & 31.9 (t)	1.18, 28H, brs 1.58, 8H, brs	22.7, 29.4–29.7
20	1.56, 2H, m	14.1 (q)	0.81, 3H, t, J = 6.6Hz	14.1

a = state its identity.

**Table 5:** The ^1^H (300 MHz) and ^13^C (75.4 MHz) data of fraction 2.

Position	δH	δC	δH	δC
	Literature ^b^		Isolated fraction	
1		179.4 (s)		178.4
2	2.28, 2H, t, J = 7.8 Hz	33.9 (t)	1.96, 4H, brs	33.4
3–20	1.19, 36H, br s	22.7, 29.1–29.7 & 31.9 (t)	1.18, 34H, brs	22.7, 29.0–29.7 31.9
21	1.56, 2H, m	24.7 (t)	1.56, 2H,t, J = 7.4 Hz	24.7
22	0.81, 3H, t, J = 3.6 Hz	14.1 (q)	0.81, 3H, t, J = 6.6 Hz	14.1

b—give its identity.

## Data Availability

The original contributions presented in this study are included in the article. Further inquiries can be directed to the corresponding author.

## Abbreviations

The following abbreviations are used in this manuscript:

ANOVA	Analysis of variance
mg GAE/g	Milligrams of gallic acid equivalents per gram of extract
T mg QE/g	Milligrams of quercetin equivalents per gram of extract
